# Nanoscale Perovskite‐Sensitized Solar Cell Revisited: Dye‐Cell or Perovskite‐Cell?

**DOI:** 10.1002/cssc.202000223

**Published:** 2020-04-17

**Authors:** So‐Min Yoo, Seul‐Yi Lee, Esteban Velilla Hernandez, Myoung Kim, Gitae Kim, Taeho Shin, Mohammad Khaja Nazeeruddin, Iván Mora‐Seró, Hyo Joong Lee

**Affiliations:** ^1^ Department of Chemistry Jeonbuk National University (JBNU) Jeonju 561-756 South Korea; ^2^ Institute of Advanced Materials (INAM) University Jaume I Avenida de Vicent Sos Baynat, s/n 12071 Castelló de la Plana Spain; ^3^ Centro de Investigación, Innovación y Desarrollo de Materiales-CIDEMAT Universidad de Antioquia UdeA Calle 70 No. 52-21 Medellín Colombia; ^4^ Group for Molecular Engineering of Functional Materials Institute of Chemical Sciences and Engineering EPFL VALAIS 1951 Sion Switzerland

**Keywords:** impedance analysis, nanostructures, perovskites, sensitizers, solid-state dye-sensitized solar cells

## Abstract

A general and straightforward way of preparing few‐nanometer‐sized well‐separated MAPbI_*x*_Br_3−*x*_ (MA=methylammonium) perovskite photosensitizers on the surface of an approximately 1 μm thick mesoporous TiO_2_ photoanode was suggested through a two‐step sequential deposition of low‐concentrated lead halides (0.10–0.30 m PbI_2_ or PbBr_2_) and methylammonium iodide/bromide (MAI/MABr). When those nanoscale MAPbI_*x*_Br_3−*x*_ perovskites were incorporated as a photosensitizer in typical solid‐state dye‐sensitized solar cells (ss‐DSSCs), it could be verified clearly by the capacitance analysis that nano‐particulate MAPbI_3_ perovskites play the same role as that of a typical dye sensitizer (MK‐2 molecule) although their size, composition, and structure are different.

## Graphical abstract

**No time to dye**: In solid‐state dye‐sensitized solar cells (ss‐DSSCs), conventional molecular dyes are replaced by well‐defined nanoscale MAPbI_*x*_Br_3−*x*_ (MA=methylammonium) perovskites, which are prepared through a simple two‐step deposition by spin‐coating low‐concentrated precursors (≤0.3 m). These nanoscale perovskite‐sensitizers give a promising initial power conversion efficiency of 7.2 % with much room left for further improvements.

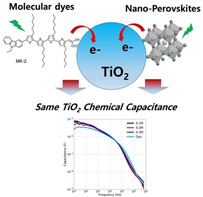

Over the last three decades, third‐generation solar cells have evolved greatly into different advanced structures by combining various nano‐ or molecular materials and using easy solution‐based processing.^1^, ^2^, ^3^, ^4^, ^5^, ^6^ In this third generation, there are mainly two different types of cells depending on the operational mechanism: one is a sensitized type, and the other is a film type. Charge generation after light absorption and then charge transportation to each electrode occur in different materials for the former (sensitized),^1^, ^2^ but in the same material for the latter (film).^3^, ^4^, ^5^, ^6^ The characteristic features of the sensitized cell come mainly from decoupling light harvesting and charge transporting, thus leading to 1) film fabrication at low cost; 2) versatile combinations of main components; and 3) effective absorption of diffuse light, making it suitable for running small devices indoors.^1^, ^2^, ^7^ The most successful example of a sensitized solar cell came from a seminal report in 1991, in which molecular dyes anchored onto the surface of mesoscopic TiO_2_ film as a photosensitizer produced significant current by injecting charges into each electron‐ and hole‐transporting material after absorbing incident light.^1^, ^8^ This dye‐sensitized solar cell (DSSC) also evolved first as a liquid type and then a solid type using solid‐state hole‐transporting material, among which the most successful one was Spiro‐OMeTAD {2,2′,7,7′‐tetrakis[*N*,*N*‐di(4‐methoxyphenyl)amino]‐9,9′‐spirobifluorene}.^1^, ^9^, ^10^ Later, semiconducting quantum dots (QDs) also attracted much attention as a replacement for typical molecular dyes toward a new class of inorganic photosensitizer in the same DSSC structure and have shown promising results recently in a liquid‐type cell after many trials.^2^, ^11^ Even in the history of recent perovskite‐based solar cell research, the first few were reported as a sensitized type cell, not a bulk film type.^12^ However, without more detailed investigations on the structures and working mechanisms of nanoscale perovskite‐sensitized cells, almost all related research efforts have been directed toward bulk perovskite films owing to the extremely high efficiency obtained in the film‐type perovskite solar cells.^5^, ^6^, ^13^

Meanwhile, molecular dye‐ or inorganic QD‐sensitized solid‐state (ss) solar cells based on mesoporous metal‐oxide films have experienced a very slow rise in overall power conversion efficiencies (PCEs) although their importance was clearly recognized in terms of using stable solid hole‐conductors relative to volatile liquid electrolytes.^9^, ^10^, 11b, 14 PCEs of typical ss‐DSSCs with Spiro‐OMeTAD as the best hole‐transporting material (HTM) have steady risen up to approximately 7.5 % over 1.0–2.0 μm thick TiO_2_ film during the last two decades since the first report of 1.8 %.^9^, ^15^ Recently, one exceptional case of a “zombie cell” was reported because it was assembled first as a liquid‐type DSSC with a Cu(I/II) redox couple and was observed to be working as a solid‐type cell after leakage of electrolyte solvent and drying only with the redox couple left inside mesoporous TiO_2_ film. This extraordinary type of cell has displayed approximately 11 % PCE after optimizations over a relatively thick TiO_2_ film composed of approximately 4 μm transparent and 4 μm scattering layers.^16^ As for QD‐sensitized solid cells, the best PCE is approximately 8 % with polymer HTMs owing to a relatively low open‐circuit voltage (*V*_oc_) from many defect‐induced recombinations at the QD itself or the interfaces with electron‐transporting material (ETM) and HTM.^17^ Overall, typical dye‐ or QD‐sensitized solid solar cells using a thin meso‐metal‐oxide film less than approximately 2.0 μm thick have reached 6–8 % PCE so far, although many efforts have been made with a powerful molecular dye or QDs as a photosensitizer situated between a meso‐ETM and solid HTM layer. Therefore, more ideal photosensitizers with stronger absorptivity and better defect tolerance are still needed for the enhanced PCEs of ss‐DSSCs. While pursuing this goal and being inspired by current perovskite bulk film‐based solar cell research, we believed that nanoscale perovskites could be a good candidate of sensitizer for ss‐DSSCs based on a thin meso‐film of metal oxides. Even when considering the very early results of perovskite solar cells and recent reports on nanoscale perovskites,^12^, ^18^ this study on nano‐perovskite photosensitizers is still in a very early stage and needs a more general preparation method for well‐defined and predictable nanoscale perovskite photosensitizers and a general working mechanism based on systematic investigations for further progress. In this study, we show a simple but effective route for preparing nanoscale MAPbI_*x*_Br_3−*x*_ (MA=methylammonium) perovskite photosensitizers on relatively thin mesoporous TiO_2_ films by using a two‐step deposition of low‐concentrated precursors. When capacitance of these samples as a function of frequency is analyzed and compared with MK‐2 sensitizer in standard ss‐DSSCs, similar capacitances are observed, pointing to the chemical capacitance of the mesoporous TiO_2_ layer. This finding indicates clearly that nano‐perovskites are working as a photosensitizer based on the same principle as the typical molecular sensitizers in DSSCs.

In the few very early results of perovskite solar cell research that were reported as MAPbI_3_‐sensitized type cells,^12^ the amount of precursor used was almost the same as that for preparing current bulk perovskite films, usually with a value of approximately 1.0 m. Soon, it was established that a more highly concentrated precursor solution (≈1.3 m) should be spin‐coated over a very thin mesoporous metal‐oxide film (<300 nm thick) for current record‐efficiency optoelectronic films.^5^, ^6^, ^19^ However, in our recent reports, it was clearly found that relatively low‐concentration precursors (Pb^2+^/MAI) are enough to prepare nanoscale MAPbI_3_ perovskites over approximately 0.65 μm thick meso‐TiO_2_ film.18a Here, as shown in Scheme 1, a very general two‐step method was suggested for preparing a few‐nanometer‐sized MAPbI_*x*_Br_3−*x*_ as a photosensitizer by depositing precursors in situ in the cell structure of ss‐DSSCs. First, lead halide (PbI_2_ or PbBr_2_) was scattered and adsorbed on the bare surface of approximately 1 μm thick mesoporous TiO_2_ film by spin coating a small aliquot of low‐concentrated (≤0.3 m) PbI_2_ (or PbBr_2_) solution (Scheme 1 b). Then, a small amount of MAI/MABr solution was dropped over the spinning PbI_2_‐ or PbBr_2_‐adsorbed TiO_2_ electrode to induce the formation of MAPbI_*x*_Br_3−*x*_ on the surface of TiO_2_ (Scheme 1 c). However, when these two low‐concentrated precursors were dissolved together in one chemical bath and spin‐coated by one‐step deposition, less homogeneous nano‐MAPbI_3_ perovskites were obtained with a lower PCE compared with the results from the current two‐step deposition when tested in the configuration of ss‐DSSC (Figure S1 in the Supporting Information). In the one‐step procedure, some aggregates could be formed more easily because the solvent (DMF) used also dissolves the deposited material better than *t*BuOH/chlorobenzene in the two‐step process, thus inducing the dissolved material to be aggregated and gathered at the upper part of the film during the spin‐coating process. Thus, the two‐step deposition is more effective in preparing well‐defined nanoscale perovskites homogeneously over meso‐metal‐oxide films in the case of using low‐concentrated precursor solutions.

**Scheme 1:**
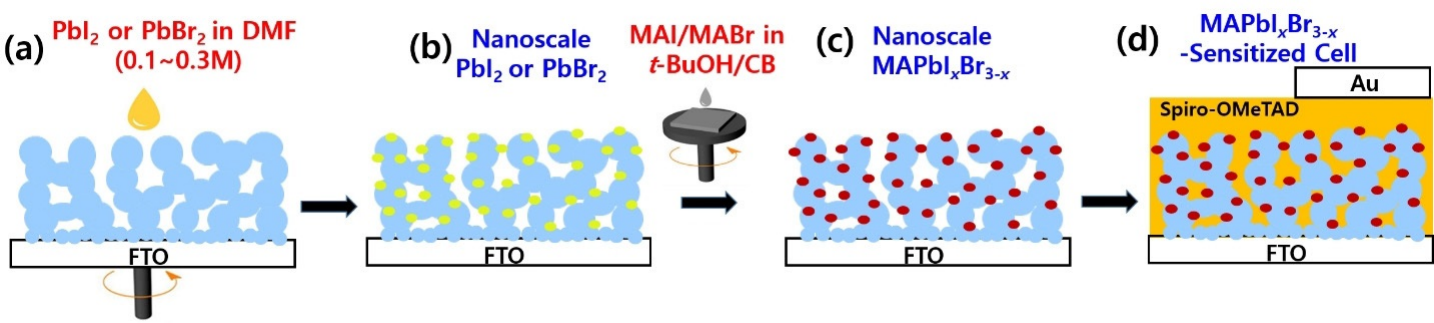
(a–c) A simplified diagram showing a two‐step deposition for preparing nanoscale MAPbI_*x*_Br_3−*x*_, and (d) a full cell structure after incorporating Spiro‐OMeTAD as a hole‐transporting layer and gold as a counter electrode, respectively.

To check the morphological status of deposited MAPbI_3_ on meso‐TiO_2_ film after following Scheme 1, electron microscopy analysis using SEM and TEM was applied to obtain images of TiO_2_/MAPbI_3_ at different magnifications. Figure 1 a, b shows a top surface and a cross‐sectional area, respectively, after spin‐coating an aliquot of 0.30 m PbI_2_ and 0.06 m MAI solution successively over approximately 1 μm thick meso‐TiO_2_ film. They look like bare TiO_2_ film without any deposits because the sizes of MAPbI_3_ deposit from relatively low concentrations of precursors (≤0.3 m) are too small to be detected in the typical magnification range of SEM. Thus, the TiO_2_ film appears to have no deposits in Figure 1 a, b. However, when the film was scanned with energy‐dispersive X‐ray spectroscopy (EDX) over the cross‐section area, the main elements (Pb and I) of MAPbI_3_ perovskites were detected to be distributed homogeneously along with an overlap of Ti from TiO_2_, which indicates the presence of nanoscale MAPbI_3_ on the surface of TiO_2_ even though it was not seen in the SEM images (Figure S2 in the Supporting Information). More directly, when the MAPbI_3_‐deposited TiO_2_ film was cut into the smallest piece possible and put over a TEM grid for imaging, a clear distribution of few‐nanometer‐sized small dots of MAPbI_3_ perovskites around TiO_2_ particles was seen in high‐resolution (HR)TEM (Figure 1 c). The deposition pattern and size of MAPbI_3_ look almost the same as those of typical SILAR (successive ionic layer adsorption and reaction)‐deposited QD sensitizers such as CdS,^20^ PbS,^21^ CdSe,11b, 22 and others^23^ on the surface of meso‐metal‐oxide films because their formation mechanism is very similar to the adsorption of the first precursor and subsequent reaction with a second one for nanoscale growth on the surface. By increasing the concentration from 0.1 to 0.3 m in the first step, the amount of nanoscale MAPbI_3_ deposited could be increased gradually as confirmed by enhanced optical density in absorbance (Figure S3 in the Supporting Information). Morphological and structural analyses by XRD (Figure S4 in the Supporting Information) clearly confirmed that nanoscale MAPbI_3_ perovskites are distributed well on meso‐TiO_2_ film. When checking both steady and time‐resolved photoluminescence, nano‐perovskite MAPbI_3_ dots on ZrO_2_ showed a blue shift of approximately 25 nm in the maximum emission peak and a faster decay of electron lifetime compared to those of a typical bulk perovskite film (Figure S5 in the Supporting Information). These could be further evidence of nanoscale perovskite formation by showing a stronger effect of confinement in a smaller nanoscale volume.^24^

**Figure 1:**
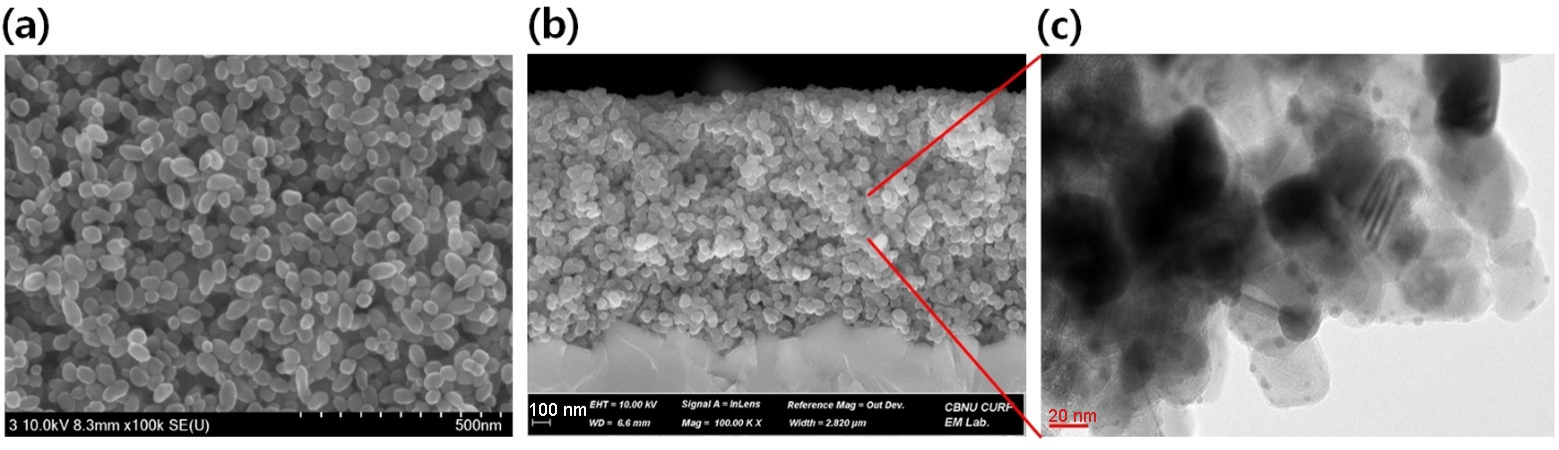
SEM images of (a) top surface and (b) cross‐section after depositing nanoscale MAPbI_3_ from 0.30 m PbI_2_, and (c) a TEM image of TiO_2_/nanoscale MAPbI_3_ from one part in (b).

These few‐nanometer‐sized MAPbI_3_ were expected to play the role of a photosensitizer comparable to molecular dye or inorganic QDs in the mesoporous film‐based sensitized solar cells reported thus far. When these nanoscale MAPbI_3_ produced by Scheme 1 were incorporated into a typical ss‐DSSC with approximately 1 μm thick TiO_2_ film, they showed approximately 4–7.2 % PCEs depending on the starting PbI_2_ concentrations used (Figure 2 and Figure S6 in the Supporting Information). With more MAPbI_3_ deposits in the nanoscale regime, much incident light is absorbed, and thus more photocurrents are induced as summarized in Table 1. The main reason for PCE enhancement was the increase of short‐circuit current (*J*_sc_) in more concentrated precursors used. The best result with 0.3 m PbI_2_ was 7.2 % under the standard 1 sun condition, comparable to the best ones from dye‐ or QD‐sensitized solid cells with a similar cell structure and materials reported so far.15b, 17b When a typical organic dye (coded as MK‐2) was tested in the same cell‐structure for comparison of the sensitizer performance, only approximately 2.6 % PCE was obtained. Moreover, the current nanoscale perovskite‐sensitized cells have not been fully optimized, and there is much room for further development toward more efficient sensitized cells. This enhancement would be possible by checking main components and their interfaces: 1) the mesoporous electron‐transporting layer of the TiO_2_ film could be adjusted to host more nano‐perovskite sensitizers effectively by controlling its thickness, pore size, and surface states; 2) a different selection of precursors and deposition routes could be used for more adsorption and charge generation; 3) a more facile infiltration of Spiro‐OMeTAD or other HTM and its intimate contact with nano‐perovskites to reduce resistance could be achieved; and 4) other optimizations at the interfaces of mesoporous films could be explored to reduce recombination.

**Figure 2:**
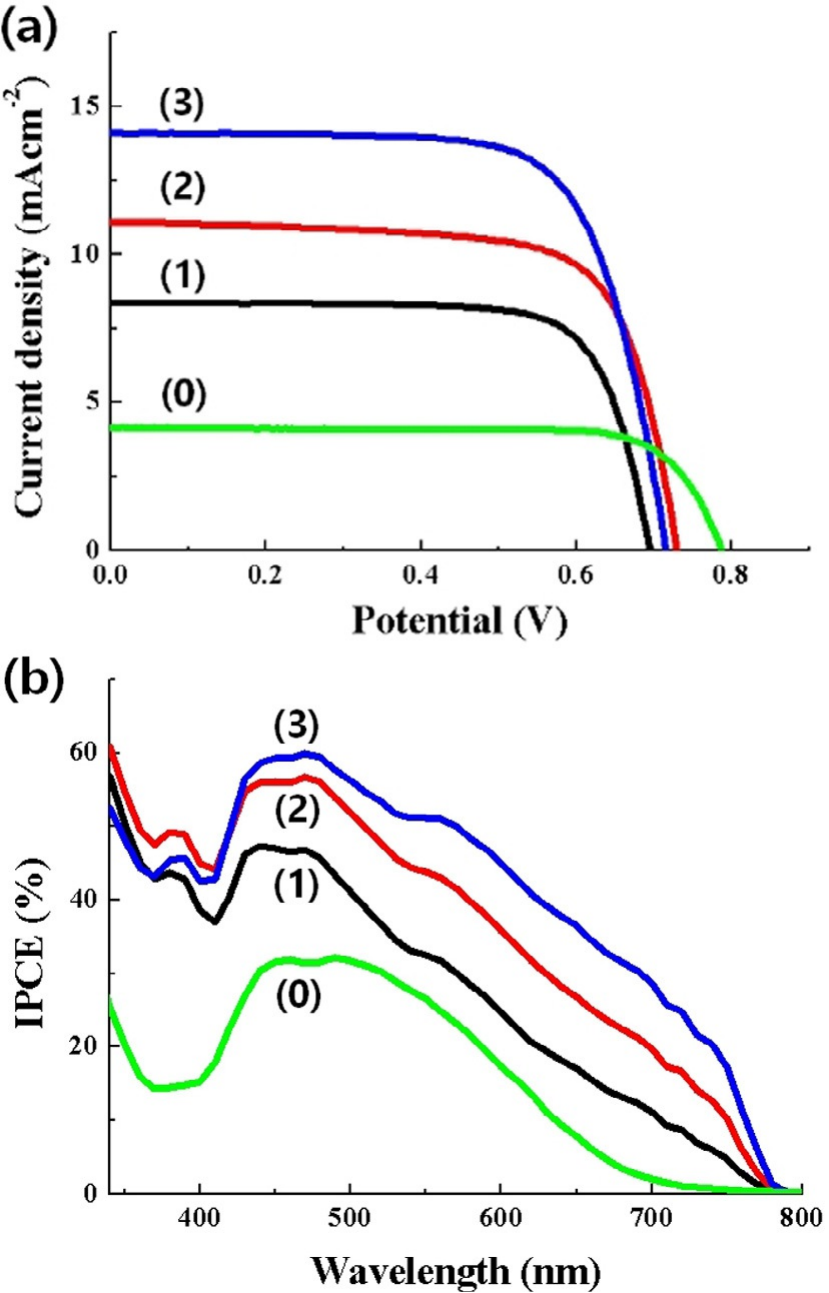
(a) Current–voltage and (b) incident photon‐to‐current conversion efficiency (IPCE) curves from the best nanoscale MAPbI_3_‐sensitized cells prepared with (1) 0.10 m, (2) 0.20 m, and (3) 0.30 m PbI_2_ precursor, respectively, along with a typical dye (MK‐2) cell (0).

**Table 1:** Main photovoltaic parameters from best nanoscale MAPbI_3_‐sensitized cells prepared by different precursor concentrations along with statistically treated values in parentheses. For comparison, the results from an organic dye (MK‐2) sensitizer are included.

Cell	*J*_sc_ [mA cm^−2^]	*V*_oc_ [V]	Fill factor	Efficiency [%]
0.1 m PbI_2_	8.36 (8.16±0.59)	0.69 (0.69±0.01)	0.76 (0.74±0.02)	4.39 (4.17±0.33)
0.2 m PbI_2_	11.09 (10.89±0.42)	0.73 (0.73±0.01)	0.72 (0.70±0.01)	5.80 (5.60±0.16)
0.3 m PbI_2_	14.10 (13.74±0.50)	0.72 (0.72±0.02)	0.71 (0.71±0.01)	7.20 (6.98±0.15)
dye (MK‐2)	4.70 (4.63±0.14)	0.79 (0.77±0.02)	0.71 (0.70±0.01)	2.64 (2.62±0.06)

To understand the working principles, impedance spectroscopy characterization was performed for three low‐concentrated precursor‐based perovskite cells along with one typical organic dye (MK‐2) cell. The same trend was observed for all analyzed samples presenting two demi‐arcs. The Nyquist plots of the impedance spectra of all the samples measured at *V*_oc_ by different light illumination conditions are depicted in Figure S7 in the Supporting Information, and the same pattern is observed. Figure 3 a, b shows the Bode plots of the measured capacitance under 0.1 sun illumination at an applied bias of 0.2 and 0.5 V, respectively. These results clearly show that all four kinds of analyzed samples present the same capacitance in a range of low and intermediate frequencies (<10^4^ Hz). It is well known that this capacitance in the case of DSSCs, such as the MK‐2‐sensitized cell, corresponds to the chemical capacitance of a mesoporous TiO_2_ layer.^25^, ^26^ This coincidence of capacitances clearly indicates that all samples work as a sensitized type, in which photo‐excited electrons in the sensitizer (dye or nano‐perovskites) are injected into the mesoporous TiO_2_ layer, raising the electron Fermi level in the TiO_2_.^27^ Moreover, photo‐excited holes are injected into the Spiro‐OMeTAD acting as a hole transporting layer. This behavior is different from that observed in bulk film‐based perovskite solar cells, for which the capacitance in the intermediate‐frequency range (10^2^–10^4^ Hz) decreases in comparison with the sensitized type by the decrease in the chemical capacitance, whereas the capacitance in the low‐frequency region (1–10^2^ Hz) increases.^28^ The percolation of carriers along the perovskite layer with low‐density band‐gap states is the origin of the decrease in chemical capacitance observed in bulk film of perovskite solar cells. In contrast, in Figure 3 a, b the same capacity is observed regardless of the amount or the type of sensitizer, indicating that this is the chemical capacitance of the common part in all samples, that is, the mesoporous TiO_2_ layer.

**Figure 3:**
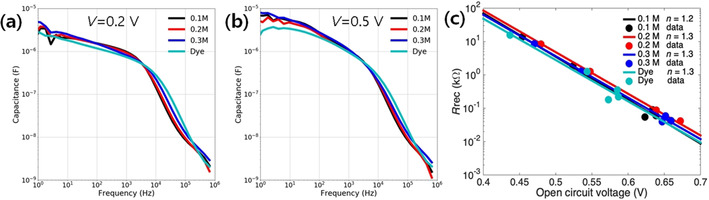
Bode plot of the measured capacitance under 0.1 sun illumination of MAPbI_3_‐sensitized cells prepared with different precursor concentrations and dye‐ (MK‐2) sensitized solar cell, at applied bias of (a) *V=*0.2 V and (b) *V=*0.5 V. (c) Recombination resistance at different open circuit potentials (varying the illumination), and the ideality factors obtained for each analyzed sample.

After understanding the working principles of the fabricated devices, it was possible to further characterize and compare the performance of the different types of devices with an analysis of the impedance spectra.^29^ The impedance spectra have been fitted using the equivalent circuits for all solid DSSCs.^25^, ^28^ The recombination resistance, *R*_rec_, for the different samples is plotted at different open circuit potentials (by varying the illumination) in Figure 3 c. Similar trends were obtained from all samples regardless of the type of sensitizer or its amount. By fitting the slope of Figure 3 c, it is also possible to obtain the ideality factor of the device, *n*, which also provides important information about the recombination mechanisms.^30^ In our case, all samples present similar *n* values of approximately 1.2–1.3, pointing to a surface ‐mediated Shockley–Read–Hall recombination.^31^

The current two‐step deposition of PbI_2_ adsorption and its reaction with MAI for a representative perovskite MAPbI_3_ could be expanded to a more general formula MAPbI_*x*_Br_3−*x*_ for modulating the absorption range over the entire visible spectrum. By adsorbing PbI_2_ or PbBr_2_ first and then applying a mixture of MAI and MABr in different ratios, different range‐sensitizing MAPbI_*x*_Br_3−*x*_ were prepared depending on the ratio of MAI and MABr in the second step. When PbI_2_ was adsorbed first and a mixture of MABr/MAI was applied with a gradual increase in the ratio, the current onset point shifted from 800 to 600 nm (Figure 4 a). In the case of initial PbBr_2_ adsorption, the onset point moved from 700 to 550 nm (Figure 4 b). Thus, by controlling the first adsorbed metal halide and the applied ratio of one or two alkyl halides, it is possible to prepare various nanoscale MAPbI_*x*_Br_3−*x*_ photosensitizers on meso‐TiO_2_ film with controlled absorption ranges. From the current results, it seems possible to prepare more versatile nanoscale perovskites, including lead‐free ones, over mesoporous films for various applications such as photosensitizers, photocatalysts, light‐emitters, and others with a tailor‐made sensitizing range by controlling the ratio of halides.

**Figure 4:**
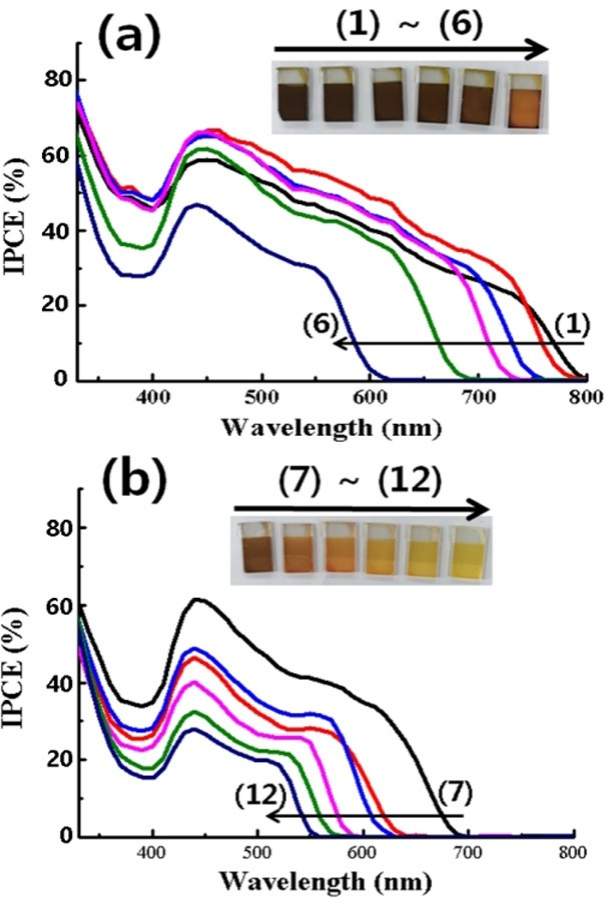
IPCE curves of nanoscale MAPbI_*x*_Br_3−*x*_‐sensitized cells from (a) PbI_2_ and (b) PbBr_2_ deposition as the first step followed by addition of a mixture of MAI and MABr in decreasing molar ratios from 5:0, 4:1, 3:2, 2:3, 1:4, and 0:5 [1–6 in (a) and 7–12 in (b)].

In summary, well‐defined and controllable nano‐MAPbI_x_Br_3−*x*_ perovskite photosensitizers could be prepared on the surface of approximately 1 μm thick mesoscopic TiO_2_ photoanode through a simple two‐step deposition of low‐concentrated precursors (≤0.3 m) in contrast to highly concentrated ones (>1.0 m) used in typical bulk films. UV/Vis absorption, XRD, photoluminescence, and SEM/TEM measurements all confirm that MAPbI_*x*_Br_3−*x*_ perovskite photosensitizers could be grown gradually on TiO_2_ film in the nanoscale regime. In a way of pursuing more ideal and powerful photosensitizers for ss‐DSSCs based on relatively thin meso‐TiO_2_ films of 1.0–2.0 μm thickness, these nano‐perovskites were incorporated and tested in ss‐DSSSc as a sensitizer instead of typical molecular dyes and showed a promising initial PCE of over 7.0 % with more optimization points left for further improvements. From impedance spectroscopic analysis, it was revealed clearly that both nano‐perovskites and molecular dyes are working as a separate photosensitizer under the same principle, in which they are only a charge generator for transfer after light absorption, not a charge accumulator for transport in ss‐DSSCs. Therefore, these well‐separated nano‐perovskites on TiO_2_ can be considered as a new dye‐like sensitizer with a composition and structure of metal halide perovskites. In addition, owing to their appropriate sizes, these nano‐perovskites could be combined with molecular dye^32^ or QDs on mesoscopic TiO_2_ film for making more efficient hybrid sensitizers to further improve the performance of ss‐DSSCs.

## Conflict of interest

*The authors declare no conflict of interest*.

## Supporting information

As a service to our authors and readers, this journal provides supporting information supplied by the authors. Such materials are peer reviewed and may be re‐organized for online delivery, but are not copy‐edited or typeset. Technical support issues arising from supporting information (other than missing files) should be addressed to the authors.

Supplementary
