# Supplementary material for: Nanoscale Perovskite‐Sensitized Solar Cell Revisited: Dye‐Cell or Perovskite‐Cell?
Source: ChemSusChem. 2020 Apr 17;13(10):2571–6. doi: 10.1002/cssc.202000223 (PMC7496478; doi:10.1002/cssc.202000223)
Supplement: Supplementary file 1 — Supplementary [file CSSC-13-2571-s001.pdf]

ChemSusChem

Supporting Information

Nanoscale Perovskite-Sensitized Solar Cell Revisited: Dye-Cell or Perovskite-Cell?

So-Min Yoo,[a] Seul-Yi Lee,[a] Esteban Velilla Hernandez,[b, c] Myoung Kim,[a] Gitae Kim,[a] Taeho Shin,[a] Mohammad Khaja Nazeeruddin,*[d] Iván Mora-Seró,*[b] and Hyo Joong Lee*[a]

Experimental

1) Materials and Preparations

A fluorine-doped tin oxide (FTO) substrate was partially etched using zinc powder and diluted HCl solution. The substrate was cleaned by taking a sequential washing with detergent, deionized (DI) water and acetone. A compact TiO_2 layer was coated by spray-pyrolysis deposition of a solution of titanium (IV) diisopropoxide bis(acetylacetonate) diluted in ethanol (1:25, v/v) at 500 ^oC and then kept for another 30 min. Onto the as-prepared compact TiO_2 layer, a mesoporous TiO_2 layer was deposited by spin-coating an aliquot of diluted TiO_2 paste, which was prepared by mixing a 1.0 g of paste, SC-HT040 from Sharechem with a 3 mL of pure ethanol and 0.02 g of polyvinylpyrrolidone (PVP, M.W.: 40000, Sigma-Aldrich), at a spinning speed of 300 rpm for 5 sec and then 1000 rpm for 20 sec. Then the substrate was heated up gradually to 500 ^oC, which made a mesoporous TiO_2 film with ca. 950 nm thickness.

To obtain nanoscale $CH_3NH_3PbI_3$ ($MAPbI_3$) perovskites onto the surface of meso-TiO_2 film electrode, different concentration of lead(II) iodide (PbI_2, 99.99%, TCI) solutions dissolved in *N*,*N*-dimethylformamide (DMF, 99.8%, Acros Organics) varying from 0.1 M to 0.3 M with 0.2 M of 4-*tert*-butylpyridine (98%, Sigma-Aldrich) as an additive were spin-coated first. After spin-coated at 2000 rpm for 30 sec, the film was dried at room temperature for 5 min and then annealed at 70 ^oC for 5 min. After that, a 0.06 M solution of MAI (Greatcell Solar) in a mixed solvent of *tert*-butanol (≥99.5%, Sigma-Aldrich): chlorobenzene (99.8%, Sigma-Aldrich) = 4:1 (volume ratio) was dropped over the PbI_2/meso-TiO_2/FTO electrode spinning at 2500 rpm. As for $MAPbI_xBr_{3-x}$, 0.3M PbI_2 or $PbBr_2$ was spin-coated first and then followed by an addition of a mixture of MAI and MABr in decreasing molar ratios from 5:0, 4:1, 3:2, 2:3, 1:4, and 0:5, respectively.

The hole transporting materials (HTM) was applied on the TiO_2/nano-perovskite surface and stayed for 30 sec before being spin-coated at 500 rpm for 10 sec first and consecutively at 2500 rpm for another 20 sec. A 70 mM spiro-OMeTAD solution was prepared by dissolving it in chlorobenzene with 56 mM of *t*BP, 14 mM of lithium bis(trifluoromethylsulphonyl)imide (Li-TFSI, 99.95%, Sigma-Aldrich) from a stock solution dissolved in acetonitrile (99.9+%, Acros Organics), and 1.5 mM of tris(2-(1*H*-pyrazol-1-yl)4-*tert*-butylpyridine) cobalt(III) tri[bis(trifluoromethane)sulfonimide] (FK-209, Greatcell Solar) from a stock solution dissolved in acetonitrile. Finally, devices were completed by thermally evaporating gold (Au) as the back contact.

2) Characterization

Photovoltaic performances were analyzed under a standard illuminating condition (AM 1.5, 100 mW cm^{-2}) using a solar simulator (Oriel, 450 W Xenon, AAA class), and the current-voltage curves were obtained using a Keithley 2400 digital source meter with a mask of 4 x 4 mm^2. The light intensity was calibrated with a Si reference cell capped by an IR-cutoff filter (KG3, KG5, Newport). The incident photon-to current efficiency (IPCE) data was measured using an IQE200B (Oriel) without bias light.

Absorbance was measured with a UV-VIS-NIR spectrophotometer (Lambda 25, Perkinelmer) and XRD measurements were done using an X'pert Pro Powder (PANalytical) under Cu Kα

radiation.

Film morphology was investigated using a scanning electron microscope (SEM, SUPRA 40VP, Carl Zeiss) and EDXS maps were recorded using an Apollo X (EDAX). The powdery TiO_2/$MAPbI_3$ was collected by scraping the film into the smallest pieces, and high-resolution transmission electron microscopy (TEM) imaging was carried out using a JEOL (JEM-2010) microscope.

Impedance spectroscopy analysis was performed using an Autolab potentiostat. In order to check the reproducibility of the measurements impedance was scanned from high to low frequency (1 MHz to 100 mHz) and then from low to high frequency (100 mHz to 1 MHz) observing coincident results and ensuring the reliability of the measurement. Impedance was measured by using different conditions: 1) changing the light intensity from the LED lamp using filters between 10 and 1000 W/m^2 and 2) changing the bias between 0.1 and 0.8 V keeping constant the light intensity at 100 W/m^2. Before any IFR measure, the open circuit voltage was established for 1 minute. Finally, all measures were fitted to equivalent circuit in order to compare the spectrums and determine the patterns. In order to reduce the influence of electrical noise in the determination of the fitting parameters, we used a global optimization process that involve a genetic algorithm and simplex method to estimate the parameters as was performed in previous works.[1] To check the linearity and reliability of the impedance measurements a double set measurements have been made for each sample, observing no significant differences between measurements for the same sample. In addition at least 2 samples, have been measured at each experimental condition (dye and perovskite concentration).

Time-resolved photoluminescence (TRPL) signals at peak wavelengths were obtained using a time correlated single photon counting (TCSPC) scheme in which an electronic module (PicoHarp 300) and a photomultiplier tube with a response time of 180 ps were employed. Laser pulses with a temporal width of 50 ps at 405 nm were used for photoexcitation at time zero. The overall response time of the scheme is roughly 200 ps and the ultimate temporal resolution can be enhanced down to 50 ps by performing a deconvolution fitting analysis. The time-dependent PL curves were fitted using a sum of two exponential functions and accordingly two time constants were yielded. Steady-state photoluminescence spectra were measured using the same picosecond laser at 405 nm and a spectrograph system (HRS-300 from Princeton Instruments).

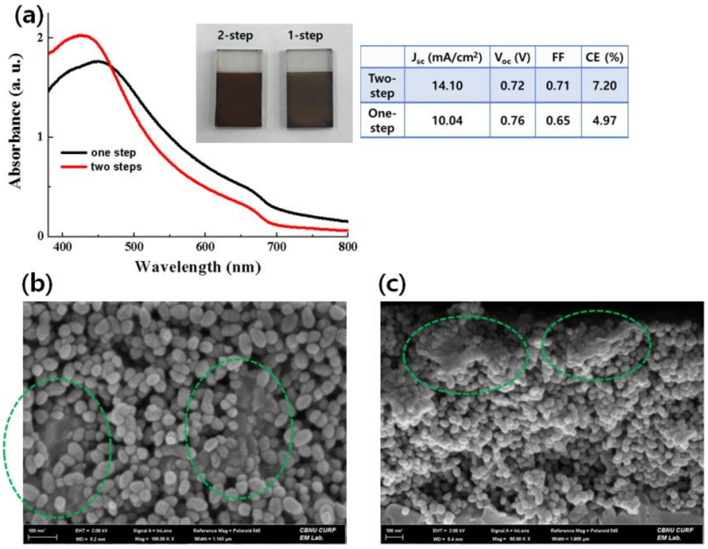

Figure S1. (a) Comparison of absorbance from TiO_2/nano-$MAPbI_3$ samples by two- and one-step deposition (one-step was done by dissolving 0.30 M PbI_2 and MAI together in DMF and applying it over FTO/TiO_2 via one-time spin-coating). SEM images of (b) top-surface and (c) cross-section of TiO_2/nano-$MAPbI_3$ sample by one-step deposition (green circle indicates some aggregates).

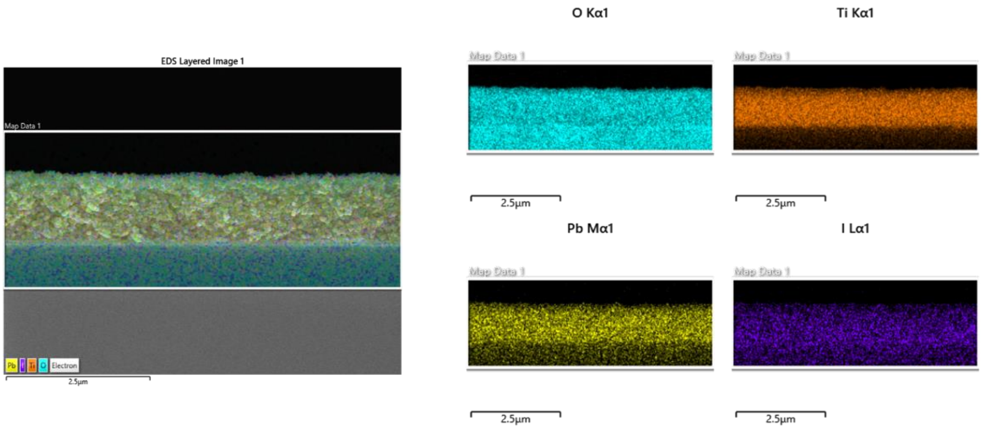

Figure S2. EDX elemental mapping of cross-sectional area of FTO glass/meso-TiO_2 film after depositing nanoscale $MAPbI_3$ by a two-step deposition using 0.30 M PbI_2.

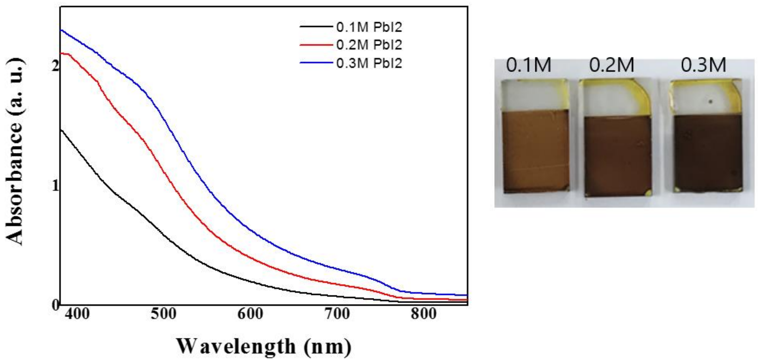

Figure S3. Absorbance and pictures of FTO glass/meso-TiO_2 electrodes after depositing nanoscale $MAPbI_3$ by a two-step deposition using 0.10, 0.20, and 0.30 M PbI_2, respectively.

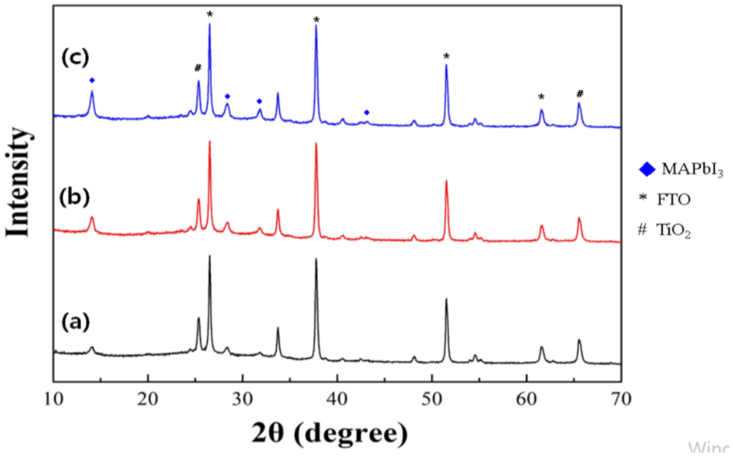

Figure S4. XRD data of FTO glass/TiO_2 electrodes after depositing nanoscale $MAPbI_3$ by a two-step deposition using (a) 0.10, (b) 0.20, and (c) 0.30 M PbI_2, respectively.

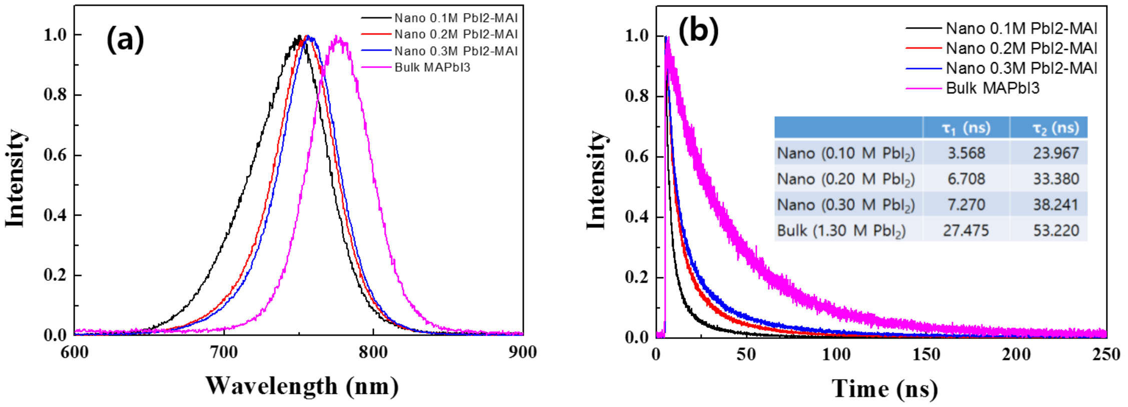

Figure S5. Steady-state (a) and time-resolved (b) photoluminescence of glass/ZrO_2 electrodes after depositing nanoscale $MAPbI_3$ by a two-step deposition using 0.10, 0.20, and 0.30 M PbI_2, respectively as well as a bulk film from1.30 M by a typical one-step deposition.

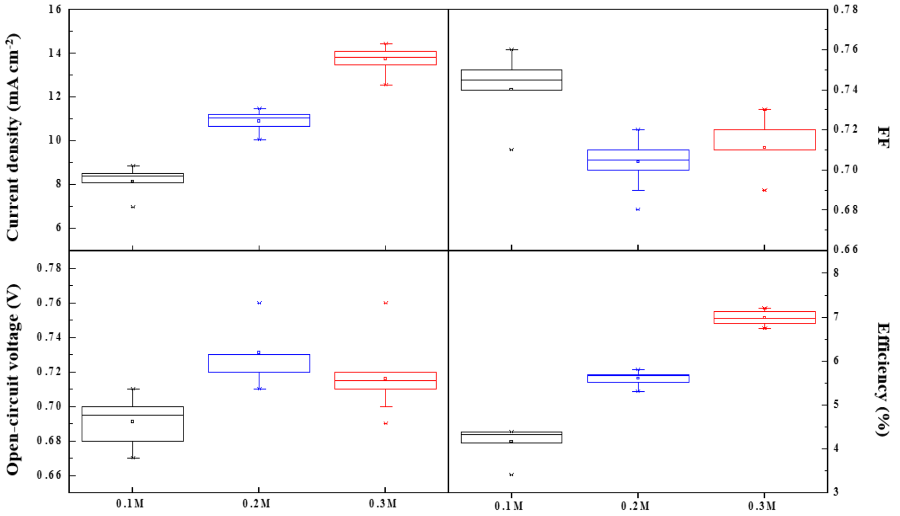

Figure S6. Statistical data set of photovoltaic parameters of nanoscale $MAPbI_3$–sensitized cells by a two-step deposition using 0.10, 0.20, and 0.30 M PbI_2, respectively.

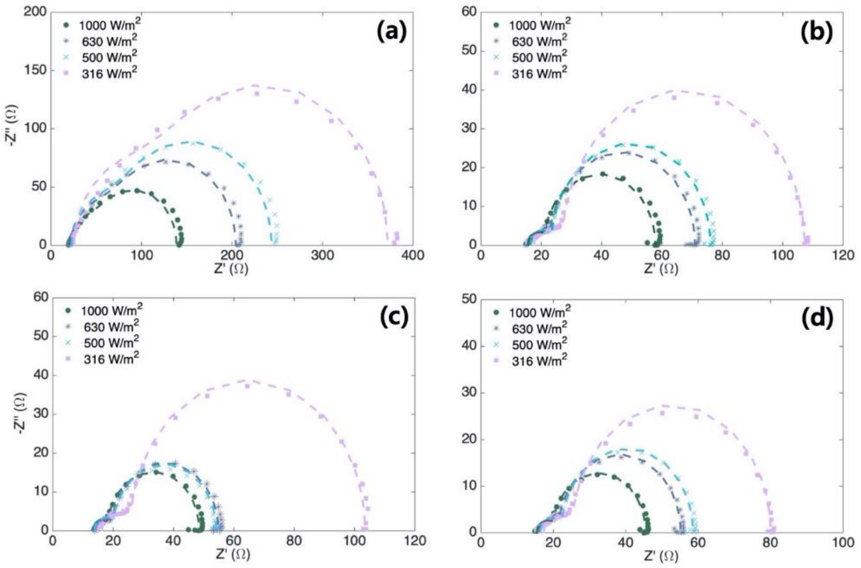

Figure S7. Nyquist plots of $MAPbI_3$–sensitized cells prepared with different precursor concentrations and dye (MK-2) sensitized solar cell, at open circuit potential by different light intensities. **(a)** Dye (MK-2) sensitized and Perovskite sensitized solar cells using different precursor concentration of **(b)** 0.1 M, **(c)** 0.2 M and **(d)** 0.3 M PbI_2. Dashed lines correspond to the fitting using the equivalent circuit describer in Ref (2).

References

(1) E. Velilla, J. B. Cano, K. Jimenez, J. Valencia, D. Ramirez, F. Jaramillo, Numerical analysis to determine reliable one-diode model parameters for perovskite solar cells. *Energies* **2018**, *11*, 1963.

(2) S.-M. Yoo, S. J. Yoon, J. A. Anta, H. J. Lee, P. P. Boix, I. Mora-Seró, An equivalent circuit for perovskite solar cell bridging sensitized to thin film architectures. *Joule* **2019**, *3*, 2535-2549.
